# Influence of Mn^2+^ and Eu^3+^ Concentration on Photoluminescence and Thermal Stability Properties in Eu^3+^-Activated ZnMoO_4_ Red Phosphor Materials

**DOI:** 10.3390/mi14081605

**Published:** 2023-08-15

**Authors:** Fan Chen, Muhammad Nadeem Akram, Xuyuan Chen

**Affiliations:** Department of Microsystems, Faculty of Technology, Natural Sciences and Maritime Sciences, Campus Vestfold, University of South-Eastern Norway, 3184 Borre, Norway; chenfan13571038693@163.com (F.C.); muhammad.n.akram@usn.no (M.N.A.)

**Keywords:** Eu^3+^/Mn^2+^:ZnMoO_4_, quantum efficiency, thermal stability

## Abstract

The integration of trivalent europium ion (Eu^3+^)-doped zinc molybdate (ZnMoO_4_) as red phosphors in next-generation solid-state lighting (SSL) is impeded by their extended electron lifetime and suboptimal thermal stability. To overcome these limitations, we propose a co-doping approach by incorporating Mn^2+^ and Eu^3+^ in ZnMoO_4_, aiming to improve thermal reversibility and reduce the lifetime of electron transitions. A series of Eu^3+^-doped ZnMoO_4_ and Mn^2+^/Eu^3+^-co-doped ZnMoO_4_ phosphor materials were synthesized via the conventional sol–gel method, and their photoluminescence properties were compared under high-temperature conditions. Experimental results indicate that the introduction of Mn^2+^ into Eu^3+^-doped ZnMoO_4_ leads to a decrease in quantum efficiency and electron lifetime, primarily attributed to defects within the crystal lattice and energy transfer from Eu^3+^ to Mn^2+^, resulting in enhanced non-radiative transitions. However, the addition of a small quantity of Mn^2+^ remarkably improves the thermal stability and reversibility of the phosphors. Consequently, this co-doping strategy presents a promising avenue for expanding the application possibilities of phosphor materials, particularly for high-power SSL applications subjected to elevated temperatures. Hence, Eu^3+^-only doped samples are well-suited for lighting applications due to their high IQE and excellent thermal stability. Conversely, Eu^3+^/Mn^2+^-co-doped samples show promise in applications that require a shorter electron lifetime and good reversibility.

## 1. Introduction

Phosphor-converted white-light-emitting diodes (PC-WLEDs) have emerged as a prominent technology in the advancement of next-generation solid-state lighting (SSL) [1,2,3,4]. However, conventional PC-WLEDs utilize a combination of blue light emitted by the diode and yellow light emitted by Y_3_Al_5_O_12_:Ce^3+^ (YAG:Ce^3+^) phosphor, resulting in a cool white light emission spectrum with a noticeable absence of the red component [5]. This deficiency limits the overall lighting quality [6,7,8]. To overcome this challenge, an effective strategy involves incorporating red-emitting phosphors into the YAG:Ce^3+^ phosphor matrix, thereby achieving warm white luminescence and improving the overall color rendering [9]. However, finding a suitable and cost-effective red-emitting phosphor presents a significant challenge [10,11,12,13,14]. Moreover, red-emitting phosphors must fulfill specific requirements, including high quantum efficiency, excellent thermal stability, high color purity, excitation spectrum compatibility with blue LED/LD sources, and avoidance of emission saturation at high light intensities (shorter electron lifetime). Unfortunately, identifying materials that satisfy all these criteria remains a formidable task [15,16]. Consequently, the exploration and development of high-quality red-emitting phosphor materials continue to pose significant scientific and technological challenges in the field of solid-state lighting.

A potential candidate for red-emitting phosphor materials is the trivalent europium ion (Eu^3+^)-doped molybdate. In these materials, the central metal ion is Mo^6+^, surrounded by four O^2−^ ions in an approximately tetrahedral configuration, which imparts exceptional structural stability [17,18]. Recent years have witnessed the investigation of various Eu^3+^-activated molybdate compounds, including CaMoO_4_ [19,20], BaMoO_4_ [21,22], SrMoO_4_ [23], ZnMoO_4_ [24], and MgMoO_4_ [25]. Nevertheless, these materials can be further enhanced by the introduction of co-doping ions to optimize their luminescence properties, such as Li^+^, Mg^+^, and K^+^ [26]. The working principle behind this phenomenon lies in the energy transfer from one RE^3+^ cation to Eu^3+^. Co-doping processes involving RE^3+^ ions serve as effective means to enhance luminescence intensity as they facilitate energy transfer from a sensitizer RE^3+^ cation to an activator RE^3+^ cation. This energy transfer mechanism is possibly enabled through various effects, including resonant energy transfer, non-radiative transition-mediated energy transfer, and quantum cutting [27].

In this study, we focused on investigating the influence of Mn^2+^ and Eu^3+^ concentrations on the photoluminescence and thermal stability properties of Eu^3+^-activated ZnMoO_4_ phosphor materials. A series of Eu_x_Zn_1−x_MoO_4_ (x = 0.05, 0.1, 0.15, 0.2) and Eu_0.1_Mn_y_Zn_0.9−y_MoO_4_ (y = 0.01, 0.05, 0.1) phosphors were synthesized. A comprehensive characterization of the photoluminescence properties of these samples was conducted, providing insights into the performance and behavior of these phosphor materials.

## 2. Materials and Methods

### 2.1. Synthesis

In this study, we have synthesized Eu^3+^- and Mn^2+^-doped zinc molybdate (ZnMoO_4_) using a sol–gel method combined with a high-temperature solid-state reaction [28,29]. The advantage of this method is the uniform doping of activator (Eu^3+^) and sensitizer (Mn^2+^) into the host material (ZnMoO_4_) at the molecular level. Raw materials used in the synthesis include citric acid C_6_H_8_O_7_ (99.5%), ammonium molybdate tetrahydrate (NH_4_)_6_Mo_7_O_24_·4H_2_O (99.0%), zinc citrate dihydrate Zn_3_(C_6_H_5_O_7_)_2_·2H_2_O (97%), manganese (II) nitrate tetrahydrate (Mn(NO_3_)_2_·4H_2_O) (97.0%), and europium (III) nitrate pentahydrate (Eu(NO_3_)_3_·5H_2_O) (99.9%). Citric acid acted as a chelating agent during the sol–gel process. The synthesis process involved the addition of zinc citrate powder, ammonium molybdate solution, europium nitrate (III) powder, and manganese nitrate (II) solution to the citric acid solution according to the stoichiometric ratio, followed by stirring using a magnetic stirrer. The container was sealed and heated to 100 °C for 12 h. The container was opened, and heating was continued to evaporate the water, resulting in the formation of a gel. Subsequently, the Eu^3+^/Mn^2+^-doped ZnMoO_4_ powder sample was obtained by calcining the gel at 800 °C for 4 h. Ceramic samples were prepared by subjecting the powder samples to a specific process. In the first step, we formed powder pellets by applying approximately 2 MPa pressure to a 1 cm^2^ sample area. In the second step, the powder pellets were subjected to calcination at 900 °C for 2 h, resulting in the formation of dense and durable luminescent ceramics.

### 2.2. Characterization

In this study, our primary focus is on investigating the electron lifetime of luminescence, determining the corresponding color coordinates of the emitted light, and assessing the thermal stability and reversibility of the sample. Edinburgh Instruments FS5 Fluorescence Spectrometers were utilized for recording electron lifetime and emission spectra. The color coordinates of the sample were analyzed using the Colorcalculator software according to the emission spectra. Thermal stability experiments were conducted using modified Fluorescence Spectrometers with a ceramic heater-equipped sample rack and a thermocouple detector pasted on the sample surface (corner) to monitor real-time sample temperature during heating. The sample was heated from room temperature to a maximum temperature, and the relative emission spectrum of the sample was tested at various temperatures. For different maximum heating temperatures, three experiments were conducted using different samples corresponding to maximum heating temperatures of 100 °C, 200 °C, and 300 °C, respectively.

## 3. Results and Discussion

### 3.1. Structure and Photoluminescence Performance

In this article, we present a comprehensive summary of the crystal structures and photoluminescence properties of EuxZn_1−x_MoO_4_ (x = 0.05, 0.1, 0.15, 0.2) and Eu_0.1_MnyZn_0.9−y_MoO_4_ (y = 0.01, 0.05, 0.1) samples, as previously reported [30]. Our analysis of the XRD diffraction patterns revealed that the crystal structures vary with the increase in x value in the Eu_x_Zn_1−x_MoO_4_ samples. When x < 0.15, the lattice structure of the sample is identified as Triclinic α-ZnMoO_4_ phase with P1 space group, while for x ≥ 0.15, the samples are a mixture of Triclinic structure P1 space group and Tetragonal structure I4_1_/a space group, where the Tetragonal structure is mainly contributed by ZnMoO_4_. We found that changes in the Eu^3+^-doping concentration did not significantly affect the host lattice bandgap in our absorption spectra analysis, which remained approximately constant at 3.5 eV. However, the host lattice bandgap decreased with increasing Mn^2+^-doping concentrations. This can be attributed to the excited energy levels of Mn^2+^ being slightly lower than those of the conductive band. We also noted that the excitation peak positions showed no shift since there was no change in the excited energy levels of Eu^3+^, irrespective of the Eu^3+^- or Mn^2+^-doping concentration. Furthermore, we found that the internal quantum efficiency of the Eu_x_Zn_1−x_MoO_4_ samples hardly changed with the change in Eu^3+^ concentration, remaining stable at around 90%. However, the increase in Mn^2+^ concentration led to a decrease in the internal quantum efficiency of the sample, owing to the energy transfer from Eu^3+^ to Mn^2+^. These findings provide valuable insights into the crystal structures and photoluminescence properties of Eu_x_Zn_1−x_MoO_4_ and Eu_0.1_Mn_y_Zn_0.9−y_MoO_4_ samples. 

### 3.2. Electron Lifetime

**Figure 1:**
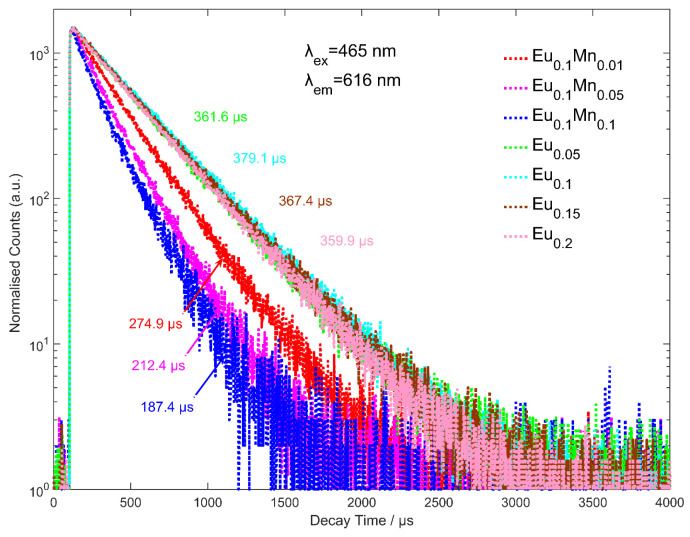
Fluorescence decay curves of Eu_x_Zn_1−x_MoO_4_ (x = 0.05, 0.1, 0.15, 0.2) and Eu_0.1_Mn_y_Zn_0.9−y_MoO_4_ (y = 0.01, 0.05, 0.1) excited at 465 nm with emission monitored at 616 nm.

Figure 1 shows the fluorescence decay curve of Eu^3+^- and Mn^2+^-doped ZnMoO_4_ powder phosphor samples for different concentrations of Eu^3+^ and Mn^2+^ ions excited at 465 nm and emission recorded at 616 nm. These decay curves can be adequately described by a second-order exponential fitting function, expressed by Equation (1) [17,31]:

(1) $$I\left(t\right)=I_{0}+A_{1}\exp\left(-t/\tau_{1}\right)+A_{2}\exp\left(-t/\tau_{2}\right)$$

where $I\left(t\right)$ represents the photoluminescence (PL) intensity at time t, $I_{0}$ is the baseline intensity, $A_{1}$ and $A_{2}$ are the pre-exponential factors corresponding to each decay component, and $\tau_{1}$ and $\tau_{2}$ represent the decay times of each component. The values of $A_{1}$, $\tau_{1}$, $A_{2}$, and $\tau_{2}$ are shown in Table 1. Using these parameters, the average electron lifetime $\tau_{ave}$ can be calculated using Equation (2):

(2) $$\tau_{ave}=\frac{A_{1}\tau_{1}{}^{2}+A_{2}\tau_{2}{}^{2}}{A_{1}\tau_{1}+A_{2}\tau_{2}}$$

The average electron lifetimes of Eu_x_Zn_1−x_MoO_4_ (x = 0.05, 0.1, 0.15, 0.2) and Eu_0.1_Mn_y_Zn_0.9−y_MoO_4_ (y = 0.01, 0.05, 0.1) are also provided in Table 2. It is obvious that the fluorescence decay times of Eu_x_Zn_1−x_MoO_4_ (x = 0.05, 0.1, 0.15, 0.2) do not exhibit significant changes with increasing Eu^3+^ concentration, reaching a maximum value when the doping concentration of Eu^3+^ ions is 10 mol%. In comparison, the decay times of Eu_0.1_Mn_y_Zn_0.9−y_MoO_4_ (y = 0, 0.01, 0.05, 0.1) samples show a noticeable decrease with increasing Mn^2+^ content, with the sample Eu_0.1_Mn_0.1_Zn_0.8_MoO_4_ exhibiting the smallest average decay time constant. The decrease in fluorescence decay time can be attributed to the cross-relaxation between Eu^3+^ and Mn^2+^ ions, but strong evidence is lacking, and this will be an important direction for future work.

### 3.3. CIE Chromaticity Coordinates

Color coordinates serve as crucial parameters in assessing the performance of phosphors, and the CIE (Commission International de L’Eclairage) chromaticity coordinates can be derived from the emission spectral data of the phosphor materials. Table 2 presents the CIE chromaticity coordinates for Eu_x_Zn_1−x_MoO_4_ (x = 0.05, 0.1, 0.15, 0.2) and Eu_0.1_Mn_y_Zn_0.9−y_MoO_4_ (y = 0.01, 0.05, 0.1) phosphors when excited at wavelengths of 395 nm, 465 nm, and 535 nm. The CIE chromaticity coordinates of Eu_x_Zn_1−x_MoO_4_ (x = 0.05, 0.1, 0.15, 0.2) exhibit a high degree of similarity and closely approximate the values of standard red chromaticity coordinates for the National Television Standards Committee (NTSC) (x = 0.670, y = 0.330). On the other hand, the CIE chromaticity coordinates of Eu_0.1_Mn_y_Zn_0.9−y_MoO_4_ (y = 0.01, 0.05, 0.1) demonstrate minor differences compared to the samples doped solely with Eu^3+^.

**Figure 2:**
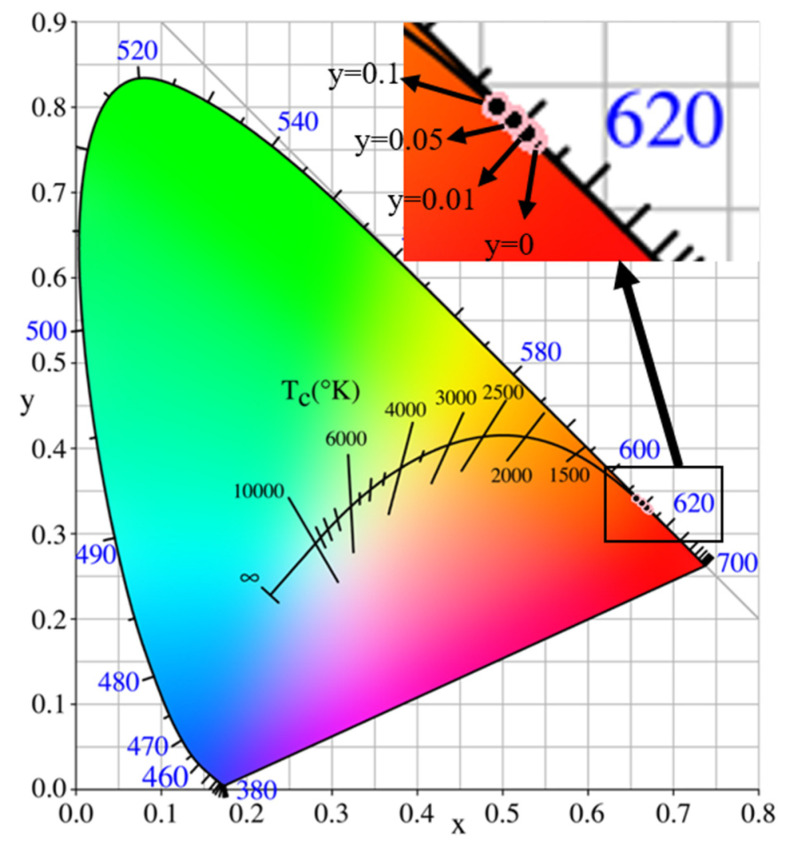
Chromaticity diagram of the CIE coordinates of Eu_0.1_Mn_y_Zn_0.9−y_MoO_4_ (y = 0, 0.01, 0.05, 0.1) samples.

Figure 2 illustrates the CIE coordinates of Eu_0.1_Mn_y_Zn_0.9−y_MoO_4_ (y = 0, 0.01, 0.05, 0.1) samples, where all the samples solely doped with Eu^3+^ coincide on the diagram. When examining Figure 2, it is evident that the CIE coordinates undergo a shift towards shorter wavelengths as the concentration of Mn^2+^ increases. The color purity can be determined using Equation (3):

(3) $$Color\;purity=\frac{\sqrt{{\left(x-x_{i}\right)}^{2}+{\left(y-y_{i}\right)}^{2}}}{\sqrt{{\left(x_{d}-x_{i}\right)}^{2}+{\left(y_{d}-y_{i}\right)}^{2}}}\times 100\%$$

The equation mentioned above calculates the color purity of a sample using the (*x*, *y*) color coordinates of the sample point, the (*x_d_*, *y_d_*) color coordinates of the illuminating light, and the (*x_i_*, *y_i_*) color coordinates of white light in the CIE diagram. For this study, the illuminating light color coordinates are (0.67, 0.32) and the white light color coordinates are (0.3101, 0.3162). The color purity of the Eu_x_Zn_1−x_MoO_4_ (x = 0.05, 0.1, 0.15, 0.2) sample is determined to be 97%.

### 3.4. Thermal Stability 

Thermal quenching is a widely observed phenomenon in phosphor materials that can significantly impact their performance under realistic operational conditions [32,33]. An accurate assessment of their thermal stability is therefore of crucial importance. In this experimental investigation, we subjected the samples to specific temperatures (100 °C, 200 °C, and 300 °C), followed by cooling to room temperature and measurement of the emission spectrum at various temperatures. Our aim was to assess the thermal stability and high-temperature reversibility of the samples. The thermal stability was determined primarily by the decrease in luminous intensity with an increase in temperature during the heating process, while high-temperature reversibility was characterized by the relationship between sample luminescence intensity and temperature during the cooling process. Our research provides new insights into the fundamental properties of phosphor materials and lays the foundation for further investigation into enhancing their performance under typical operating conditions.

**Figure 3:**
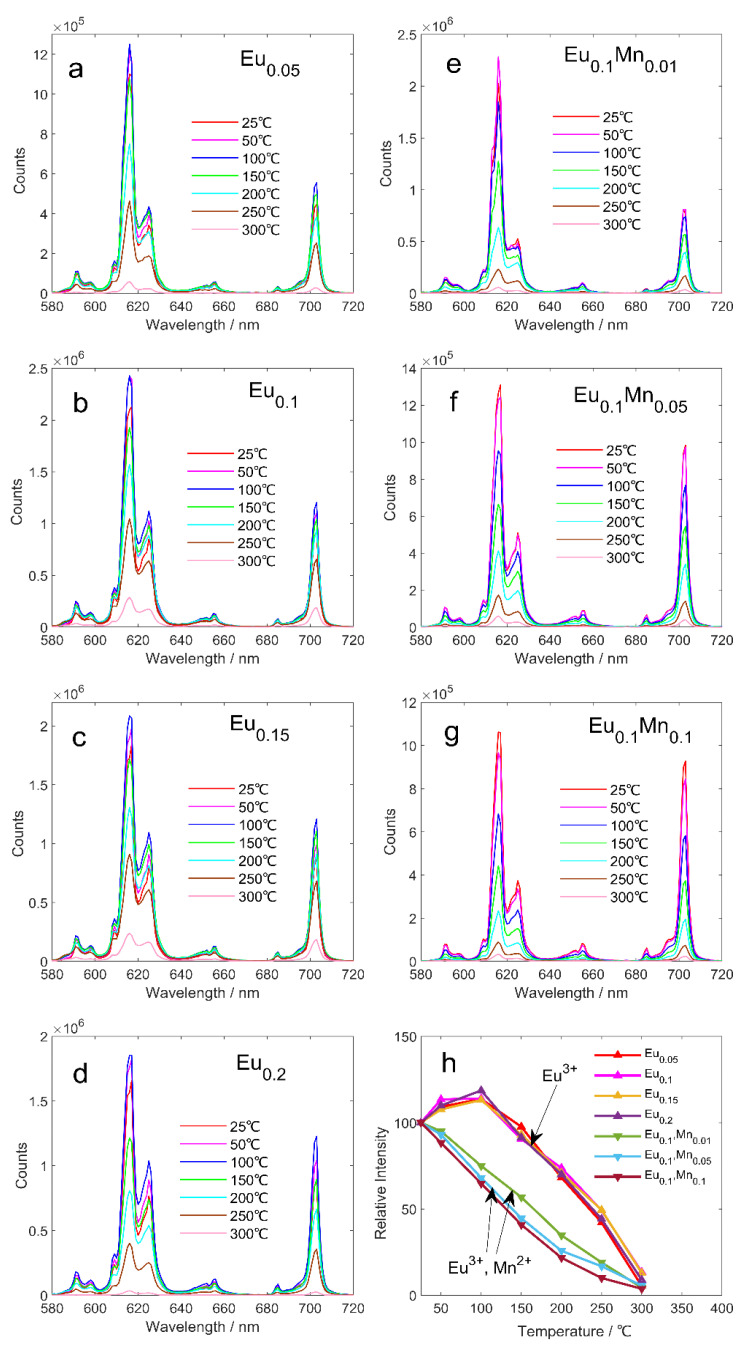
Comparison of the effects of Eu^3+^ and Mn^2+^ concentrations on the thermal stability of the samples (heated to 300 °C); (**a**–**d**) corresponds to EuxZn_1−x_MoO_4_ (x = 0.05, 0.1, 0.15, 0.2), (**e**–**g**) corresponds to Eu_0.1_Mn_y_Zn_0.9−y_MoO_4_ (y = 0, 0.01, 0.05, 0.1), comparison of all samples after spectral integration in (**h**).

The present study investigates the temperature-dependent luminescent properties of Eu^3+^/Mn^2+^-co-doped ZnMoO_4_ phosphors. The relative emission spectra of the samples were recorded over a temperature range of 25–300 °C, and the relative luminous intensities were integrated to obtain a line chart displaying the thermal stability of the samples, as shown in Figure 3. The results reveal that all samples doped with Eu^3+^ (without Mn^2+^) exhibit similar thermal stability, with the relative luminous intensity initially increasing and then decreasing as the temperature increases. At 100 °C, the relative luminous intensity reaches a peak value, which is 1.2 times that at room temperature. This phenomenon can be attributed to the increased probability of electron transition from the ground state to the excited state, leading to an increase in emission intensity due to molecular motion [34]. However, as the temperature continues to rise, the relative luminous intensity weakens, and at 300 °C, it is almost completely quenched, owing to the energy provided by the high-temperature environment that drives non-radiative transition and heat conversion, leading to thermal quenching.

Interestingly, for the samples co-doped with Eu^3+^ and Mn^2+^, the relative luminous intensity decreases with the increase in temperature and drops by approximately 50% of the room temperature at 150 °C. The addition of Mn^2+^ changes the charge transfer band of the sample, thereby lowering its energy band and bringing it closer to the excited state, reducing the thermal activation energy of the samples. Notably, the addition of Mn^2+^ alters the relative luminous intensity of the sample from enhancement to weakening in the temperature range from room temperature to 100 °C, representing a unique feature of these co-doped phosphors. These findings provide crucial insights into the thermal stability and potential applications of Eu^3+^/Mn^2+^-co-doped ZnMoO_4_ phosphors in the field of optoelectronics.

**Figure 4:**
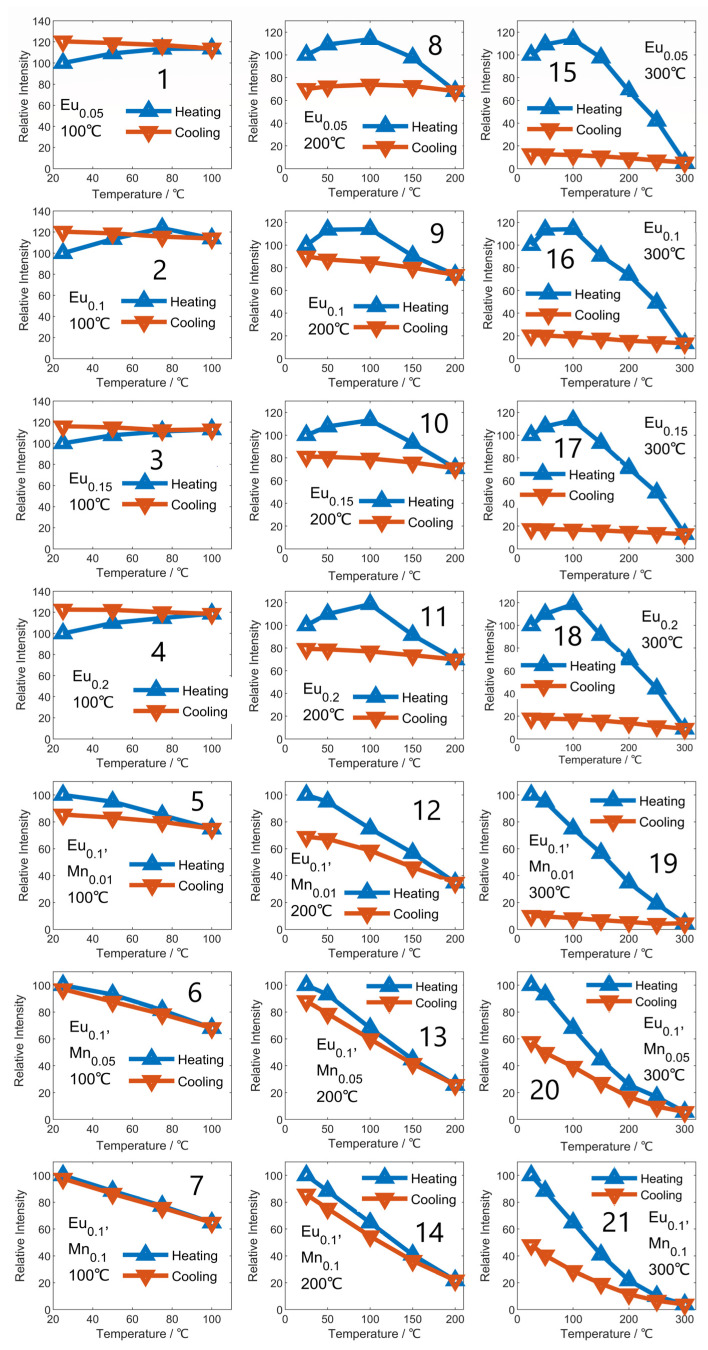
The relationship between emission intensity and temperature during the heating and cooling processes (heated to 100 °C, 200 °C, and 300 °C separately); (**1**–**7**) corresponds to all samples heated to 100 °C, (**8**–**14**) corresponds to all samples heated to 200 °C, (**15**–**21**) corresponds to all samples heated to 300 °C.

This study investigated the reversibility of Eu^3+^-doped samples under varying temperature conditions using relative luminous intensity as the observed parameter. Upon heating the sample to 100, 200, and 300 °C and subsequent cooling to room temperature, the researchers observed changes in the relative luminous intensity of the specimen, as shown in Figure 4. The results showed that the Eu^3+^-doped sample’s relative luminous intensity remained unchanged during cooling after heating to 100 °C while being higher than the standard intensity at room temperature, indicating improved luminous properties from defect annealing. However, when the sample was heated to 200 °C and 300 °C, the relative luminous intensity remained constant and consistent with the highest temperature during the cooling process [35]. This suggests that high temperatures irreversibly damage Eu^3+^-doped samples, making them unsuitable for working and storage environments with severe temperature fluctuations. Thus, understanding the temperature-induced irreversible damage in Eu^3+^-doped samples is essential for their practical use, especially where such samples are expected to face sudden temperature changes [36,37]. 

The current study investigated the impact of Mn^2+^ introduction on the temperature reversibility of Eu^3+^-doped samples. The relative luminescence intensity of the samples increased with decreasing temperature during cooling, indicating improved reversibility due to Mn^2+^ co-doping [38]. The degree of recovery of relative luminescence intensity was found to be directly related to the Mn^2+^ content and inversely related to the highest temperature experienced by the sample. Specifically, when heating and cooling the same Eu_0.1_Mn_0.05_ sample to different temperatures, the recovery of luminous intensity varied: heating to 100 °C resulted in a luminous intensity recovery from 80 to 90, while heating to 200 °C led to a recovery from 40 to 70, and heating to 300 °C did not result in any recovery. Thus, our results suggest that Mn^2+^ co-doping enhances the high-temperature reversibility of Eu^3+^-doped samples, with the strength of this reversibility being both proportional to the Mn^2+^ content and inversely proportional to the highest temperature experienced by the sample [39]. 

## 4. Conclusions

In conclusion, the addition of Mn^2+^ to the Eu_0.1_Zn_0.9_MoO_4_ sample resulted in a decrease in electron lifetime. A shorter electron lifetime in a sample signifies a higher excitation light power saturation threshold. Hence, the Eu^3+^/Mn^2+^-co-doped samples exhibit promising potential in applications with very high excitation light power, surpassing the capabilities of Eu^3+^-only doped samples.

Thermal stability experiments indicated that the Eu^3+^-only doped samples exhibited superior performance compared to the Eu^3+^/Mn^2+^-co-doped samples. Conversely, the co-doped samples demonstrated enhanced reversibility. Hence, Eu^3+^-only doped samples are well-suited to lighting applications due to their high IQE and excellent thermal stability. Conversely, Eu^3+^/Mn^2+^-co-doped samples show promise in applications that require a shorter electron lifetime and good reversibility.

Overall, this study highlights the potential of Eu^3+^-only doped and Eu^3+^/Mn^2+^-co-doped samples in different applications based on their unique characteristics. The findings contribute to the understanding of the optical properties and performance of these phosphor materials, paving the way for their utilization in diverse fields. Further exploration and optimization of these materials can unlock their full potential for various practical applications.

## Figures and Tables

**Table 1:** The parameters of fitting fluorescence decay curves of Eu_x_Zn_1−x_MoO_4_ (x = 0.05, 0.1, 0.15, 0.2) and Eu_0.1_Mn_y_Zn_0.9−y_MoO_4_ (y = 0.01, 0.05, 0.1) excited at 465 nm with emission monitored at 616 nm.

Samples	*A*_1_	*τ*_1_/µs	*A*_1_	*τ*_1_/µs	*τ_ave_*
x = 0.05	5537.2	334.9	506.1	542.0	361.6
x = 0.1	4879.9	355.4	1755.2	432.8	379.0
x = 0.15	2773.5	329.1	1841.6	413.3	367.4
x = 0.2	3963.1	324.0	1391.5	435.8	359.9
y = 0.01	1744.4	216.1	1877.8	312.6	274.9
y = 0.05	999.6	144.2	1342.4	242.6	212.4
y = 0.1	482.6	109.3	782.0	212.2	187.4

**Table 2:** CIE chromaticity coordinates of Eu_x_Zn_1−x_MoO_4_ (x = 0.05, 0.1, 0.15, 0.2) and Eu_0.1_Mn_y_Zn_0.9−y_MoO_4_ (y = 0.01, 0.05, 0.1) samples excited at 395 nm, 465 nm, and 535 nm.

Samples	395 nm		465 nm		535 nm	
	x	y	x	y	x	y
x = 0.05	0.6722	0.3276	0.6711	0.3286	0.6634	0.3363
x = 0.1	0.6725	0.3272	0.6719	0.3279	0.6682	0.3315
x = 0.15	0.6723	0.3275	0.6714	0.3284	0.6678	0.3319
x = 0.2	0.6717	0.3280	0.6709	0.3289	0.6667	0.3330
y = 0.01	0.6674	0.3324	0.6689	0.3308	0.6595	0.3401
y = 0.05	0.6523	0.3473	0.6643	0.3354	0.6472	0.3524
y = 0.1	0.6360	0.3636	0.6577	0.3419	0.6264	0.3730

## Data Availability

More research data are available from the authors on request.
